# Quantum dots in periodontology: emerging promise and translational challenges

**DOI:** 10.3389/fdmed.2026.1871912

**Published:** 2026-06-24

**Authors:** Richik Chakraborty, Nina Shenoy, Rahul Bhandary

**Affiliations:** Nitte (Deemed to be University), AB Shetty Memorial Institute of Dental Sciences (ABSMIDS), Department of Periodontology, Mangalore, India

**Keywords:** carbon dots, dentistry, local drug delivery, nanotechnology, periodontology, quantum dots

## Abstract

Periodontitis is a chronic inflammatory disease driven by microbial dysbiosis, resulting in irreversible destruction of the periodontal ligament and alveolar bone. Conventional therapies, including mechanical debridement and local drug delivery, frequently fail to achieve adequate outcomes in advanced disease due to poor biofilm penetration, limited site-specificity, and the inability to modulate the host immune microenvironment. Quantum Dots (QDs) are semiconductor nanocrystals measuring 1–10 nm, which possess unique size-dependent photoluminescence, high photostability, broad excitation profiles, and versatile surface functionalization, properties that have not yet been systematically evaluated in the context of periodontology. This review evaluates QD applications across four domains: diagnostics and bioimaging, targeted therapeutics and local drug delivery, tissue engineering and regeneration, and dental implantology. In diagnostics, QDs enable ultrasensitive detection of salivary and crevicular inflammatory biomarkers, real-time pathogen imaging, and integration into wearable point-of-care platforms. Therapeutically, they facilitate photodynamic antimicrobial therapy, stimuli-responsive drug release, and improved bioavailability of agents such as curcumin and metformin. In regeneration, they promote osteogenic stem cell differentiation and immunomodulation of the local inflammatory microenvironment. On implant surfaces, they enhance antibacterial activity and osseointegration. Despite this breadth, clinical translation remains constrained by cytotoxicity of heavy-metal-based variants, physicochemical instability in the oral environment, and the absence of long-term *in vivo* data and harmonized regulatory pathways. QDs especially emerging carbon-based variants represent a scientifically promising nanoplatform for precision periodontal care, but bridging the gap from bench to chair will require standardized synthesis, rigorous safety profiling, and well-designed translational studies.

## 1 Introduction

Quantum Dots (QDs) are nanocrystals with zero dimensions, usually made from Group II-VI or III-V semiconductor materials (1), and typically measure 1–10 nanometers across. QDs have unique electronic properties and can be tailored for various uses, making them valuable for biomedical applications (2). The color of light they emit depends on their size: smaller QDs emit shorter wavelengths, while larger QDs emit longer ones (3). They resist photobleaching, have stable optics, and boast a high quantum yield, supporting extended tracking in biological systems (4, 5). QDs can be excited by a wide range of light but emit light in a narrow, symmetric spectrum, making it easier to detect multiple biomarkers simultaneously in multiplex imaging (1). Their large surface area relative to volume allows QDs to be coated with specific molecules, such as antibodies or peptides, for targeted recognition and delivery (4). Carbon-based QDs are attracting greater interest because they are more compatible with living systems, less toxic, and more readily soluble in water (6).

QDs serve as contrast agents in magnetic resonance imaging (MRI) and photoacoustic imaging, and as photosensitizers in photodynamic therapy (PDT) for cancer, thereby integrating diagnostic imaging with therapeutic delivery (7). Applications of QDs in dentistry include diagnostics, where newer devices like zinc oxide quantum dots (ZnOQDs) containing wearable mouthguards detect volatile sulphur compounds (VSCs), in restorative dentistry their use in composite resins as fillers to promote antibacterial activity against *Streptococcus mutans* (2), in tooth whitening procedures through reactive oxygen species (ROS)-mediated stain degradation by copper-doped carbon dots (CDs) (8), and in tissue engineering, its promotes osteogenic and odontogenic differentiation of stem cells, through pathways such as Wnt/*β*-catenin (2, 9).

The removal of dysbiotic biofilms, which destroys the periodontal ligament and alveolar bone, requires conventional mechanical debridement and drug delivery. However, these methods are often ineffective in deep pockets. Nanomaterials can access these sites and provide sustained local action (10, 11). To address the limits of conventional therapy, nanotechnology in periodontology is emerging (12). Beyond therapy, QD-based nanodiagnostics enable early detection of tissue damage by identifying biomarkers such as MMPs and IL-1β at very low concentrations (11). They also help modulate the host by reducing oxidative stress, suppressing pro-inflammatory cytokines, and promoting macrophage polarization toward a regenerative phenotype (13, 14), as well as support targeted bone regeneration by delivering therapeutics to inflamed sites and aiding stem cell-mediated tissue repair (11). Despite this expanding body of preclinical literature, there is currently no dedicated synthesis that maps the full mechanistic and translational landscape of QDs specifically within the domain of periodontology. Existing reviews either address nanomaterials broadly in dentistry or focus on isolated applications such as drug delivery or bioimaging, leaving a gap in the integrated understanding of how QDs can simultaneously address diagnostic, therapeutic, regenerative, and implant-related challenges unique to the periodontium. The aim of the review is to fill this gap by comprehensively evaluating current and emerging applications of QDs in periodontology, characterizing their mechanisms of action, and highlighting the key translational barriers that continue to limit their adoption in routine clinical practice.

## 2 Applications of QDs in advanced periodontal diagnostics and bioimaging

QDs are suitable for high-resolution multiplex imaging and ultrasensitive biosensing in the periodontal microenvironment and are used in advanced periodontal diagnostics and bioimaging (5).

### 2.1 High-resolution pathogen and biofilm bioimaging

Single-cell resolution imaging of bacteria using QDs in a mature biofilm enables detailed assessment of its architecture and interbacterial relationships (3). Innovative carbonized polymer dots (acidophilic dual-emission fluorescent carbonized PDs) leverage the metabolic properties of oral infections by demonstrating pH-dependent fluorescence behaviors, allowing clinicians to distinguish between acid-producing cariogenic species (e.g., *Streptococcus mutans*) and major periodontal pathogens (e.g., *Porphyromonas gingivalis*) in real-time (6). The dense extracellular polymeric matrix of oral biofilms is often inaccessible to conventional dyes; some CDs can penetrate this matrix and image the microorganisms (6, 15). Beyond imaging, ornidazole-based carbon quantum dots function as fluorescent traceable bioprobes, which offers as a novel diagnostic strategy for real-time tracking of *Porphyromonas gingivalis* (16).

### 2.2 Bioimaging of periodontal pathology and malignancy

QDs act as high-performance contrast agents and can be employed for early, precise detection of molecular changes in oral squamous cell carcinoma (1, 4). They can be conjugated to monoclonal antibodies targeting tumor-specific markers such as CEA, CA125, or EGFR (1). Near-infrared–emitting QDs allow deeper tissue penetration with reduced light scattering and absorption. This supports three-dimensional imaging of living tissues (7, 17). They are therefore useful for longitudinal monitoring of scaffold integration and cellular activity during periodontal bone repair (17).

### 2.3 Biosensing of inflammatory biomarkers

QDs are highly effective for molecular detection of periodontal disease before lesion progression because of their high surface-to-volume ratio, which allows extensive functionalization with biorecognition ligands (5, 11). QD-based biosensors can quantify inflammatory biomarkers in saliva and gingival crevicular fluid, including matrix metalloproteinases and pro-inflammatory cytokines, with sensitivities in the nanomolar to picomolar range (2). More advanced platforms, such as carbon QDs encapsulated within metal-organic frameworks, improve stability in the salivary environment while enhancing analyte capture and fluorescent signal amplification (17). DNA-templated QDs have enabled rapid detection of biomarkers such as interleukin-8 (IL-8) in oral fluids, a critical marker for both inflammation and oral cancer, and upconversion nanoparticle-based systems for electrochemical and point-of-care uses (1, 11).

### 2.4 Wearable systems and artificial intelligence integration

Personalized periodontal medicine is advancing with new, non-invasive wearable diagnostic devices. AI-based image analysis can interpret the patterns these devices detect. This allows for automated and accurate identification of periodontal inflammation (2, 11). Wearable sensors with MoS₂ QDs can analyze exhaled breath markers, such as NO₂. This may help detect caries and periodontitis early (2).

## 3 Applications of QDs in targeted therapeutics and local drug delivery

QDs achieve both precise site targeting and controlled drug release, shifting treatment from passive drug administration to a dual-function system (19).

### 3.1 Architectural strategies for local drug delivery

There are several ways to incorporate QDs into drug delivery systems. Drugs or biomolecules can be directly linked to the QD surface by covalent or non-covalent bonds. This is useful in photodynamic therapy, where *in situ* therapeutic activation occurs via energy transfer from the QD to the photosensitizer, without actual drug release (20). Alternatively, QDs may be encapsulated in hybrid carriers such as polymeric nanoparticles, liposomes, or metal-organic frameworks, which improve stability, protect from photobleaching, and allow controlled loading and release of therapeutic agents (18, 20). Graphene quantum dots (GQDs) are utilized in photodynamic therapy as efficient photosensitizing agents and combined GQD- curcumin platform creates a synergistic effect that enhances antimicrobial photodynamic therapy (aPDT) which significantly reduces viability and biomass of perio-pathogens (21). Periodontal pockets have irregular shapes. Stimuli-responsive hydrogels with QDs adapt to these tissues and gradually degrade in response to local triggers such as acidic pH or bacterial enzymes, enabling localized, on-demand drug release (13, 22).

### 3.2 Targeted therapeutics and the “dual-logic” strategy

QDs functionalizing their surface with ligands creates a dual action system consisting of two components- guidance and therapeutic activity. The guidance component helps the nanoplatform to localize precisely at the disease site; for example, Curcumin–Alendronate carbon dots (Cur-Alen CDs) use alendronate-mediated affinity for hydroxyapatite and osteoclast-rich bone surfaces, while antibody-conjugated QDs can target markers such as CEA or EGFR in oral squamous cell carcinoma (1, 18). After doing so, the therapeutic component exerts its biological effect, as seen with Cur-Alen CDs, which show enhanced antioxidant and anti-inflammatory activity, reduce cytokines such as TNF-α and IL-1β, and help restore the altered bone microenvironment (18).

### 3.3 Applications in enhancing bioavailability

Many therapeutic agents, like curcumin, have their clinical utility restricted due to poor water solubility and instability, and QD-based nanodelivery enables us to overcome this (15, 23). Curcumin, when loaded onto polyethylene glycol (PEG)- functionalized carboxylated graphene QDs, markedly improves its solubility and can accumulate in bacterial cell walls, enabling rapid photoactivated antimicrobial action (23). More recent approaches also use therapeutic drugs themselves as carbon precursors for synthesizing functional CDs. Metformin-derived carbon dots can activate osteogenic signaling pathways and restore stem cell function under inflammatory conditions, while melatonin-derived carbon dots show strong ROS scavenging activity, making them promising for periodontitis management (8, 24).

## 4 Applications of QDs in periodontal tissue engineering and regeneration

Carbon-based QDs can actively support stem cell differentiation, influence host immune responses, and act as trackable carriers for regenerative molecules (2, 14).

### 4.1 Stimulation of stem cell proliferation and differentiation

QDs improve the regenerative capacity of seed cells like human periodontal ligament stem cells and bone marrow stromal cells (2). GQDs and graphene oxide QDs (GOQDs) have shown enhanced osteogenic differentiation by upregulating markers like alkaline phosphatase, RUNX2, and osteocalcin (7, 9), as well as fiber-related markers such as COL-I and Scleraxis (25) and also promoting the formation of mineralized nodules in an inflamed environment (26). These effects are driven through signaling pathways, including Wnt/*β*-catenin, PI3 K/AKT, ERK/AMPK, and BMP2/SMAD5/RUNX2, which collectively help restore the osteogenic potential of stem cells and accelerate periodontal regeneration (9, 27, 28).

### 4.2 Mitochondrial and epigenetic regulation

Studies suggest that QDs can regulate periodontal regeneration even at the organelle and epigenetic levels. GOQDs promote mitochondrial fusion and inhibit fission, thereby shifting cellular metabolism toward oxidative phosphorylation and supporting osteogenic differentiation of periodontal ligament stem cells (26). By influencing KDM4B, epigenetic modulation is achieved using yam-derived carbon dots, which regulate osteoblast proliferation and differentiation under inflammatory conditions, thereby enhancing bone defect repair (29).

### 4.3 Osteoimmunomodulation and microenvironment remodeling

Successful periodontal regeneration requires not only the stimulation of stem cells but also the conversion of the local environment from a pro-inflammatory to a pro-healing state (14). In this regard, several CDs have been shown to promote macrophage polarization from the inflammatory M1 phenotype towards the regenerative M2 phenotype, thereby enhancing tissue repair, angiogenesis, and growth factor release (7, 14). These nanomaterials also act as potent antioxidants or nanozymes, scavenging excess ROS and thereby reducing oxidative stress–mediated alveolar bone loss (8, 19). In addition, certain carbon QDs can modulate T-cell responses by promoting their proliferation while inhibiting differentiation into pro-inflammatory phenotypes (24). This dual action contributes to the attenuation of inflammation and the preservation of periodontal tissue integrity (19).

### 4.4 *In vivo* regenerative outcomes

QD- based therapies when applied in animal studies (rats and mice) have demonstrated reduced periodontal bone loss, reflected by a decrease in the decrease in cementoenamel junction to alveolar bone crest distance, while micro-CT and histological findings show higher bone volume, denser trabecular architecture, and better collagen fiber organization when treated with QD- modified stem cell sheets or hydrogel helping us acknowledge its regenerative benefits (19, 22, 24).

Table 1 summarizes the major application domains of QDs in periodontology, including diagnostics, antimicrobial delivery, intrinsic bactericidal activity, adjunctive photodynamic therapy, bone regeneration, and periodontal ligament regeneration, along with their respective QD or carbon dot subtypes, primary mechanisms, and key signaling targets.

**Table 1:** Summary of quantum dot applications and mechanistic insights in periodontology.

Application domain	QD/CD type	Primary mechanism of action	Targeted outcome	Key signaling/target
Advanced Diagnosis	Conjugated QDs	High-sensitivity fluorescence imaging (90% sensitivity)	Early detection of pathology and molecular changes	Inflammatory biomarkers (IL-6, TNF-, MMPs) (4, 30)
Antimicrobial Delivery	TCDs, MCDs	Targeted nanocarrier penetration of protective matrix	Enhanced local antibiotic efficacy against *P. gingivalis*	Biofilm matrix penetration (31)
Intrinsic Bactericide	Carbon Quantum Dots (CQDs)	High positive surface charge (-potential mV)	Broad-spectrum membrane disruption and cell death	Bacterial membrane integrity (14)
Adjunctive Therapy	GQD-Cur	Photosensitizer generation of Reactive Oxygen Species (ROS)	Eradication of mixed biofilms and prevention of re-establishment	Pathogenic gene expression (*rcpA, fimA, inpA*) (6)
Bone Regeneration	CQDs (Metformin)	Upregulation of osteogenic differentiation markers (OCN, Runx2)	Repair of alveolar bone defects	ROS-mediated MAPK; PERK-eIF2-ATF4; EPK/AMPK pathways (32)
PDL Regeneration	Graphene Oxide QDs (GOQDs)	Modulation of internal cellular energy/fate mechanism	Promotion of mitochondrial fusion; enhanced hPDLSC differentiation	Mitochondrial dynamics (Fusion/Fission balance) (26)

## 5 Applications of QDs in dental implants

Primary applications include enhancing antibacterial activity, promoting osseointegration, and serving as vehicles for controlled drug delivery.

### 5.1 Antibacterial and anti-biofilm applications

GQDs incorporated into titanium dioxide (TiO_2)_ nanorods on titanium dental implants show an antibacterial activity against *Streptococcus mutans* (33). Boron-doped carbon quantum dots attached to titanium alloys exhibit antibacterial effects against both gram-positive and gram-negative bacteria (34). Chiral carbon dots (CCDs) can be incorporated into pH-sensitive poly(lactic-coglycolic) acid (PLGA) coatings to provide a medium-term controlled release (up to 28 days), specifically triggering higher release rates in the acidic environments (pH ∼3.0) characteristic of inflammatory and infection sites (35).

### 5.2 Enhanced osseointegration and bone regeneration

In animal models, implants modified with GQDs promoted significantly new bone formation even in the presence of existing infections (33). GQDs deposited on TiO_2_ nanotubes have been shown to significantly enhance adhesion, proliferation and osteogenic differentiation of bone marrow-derived mesenchymal stem cells (BMSCs) (36).

### 5.3 Bioimaging and diagnostic sensing

The intrinsic photoluminescence of CDs and GQDs is used for tracking cellular interactions with the implant surface in real-time (37), and detecting fluctuations in ions or proteins at the implant site, which might help to monitor healing or early-stage infection (38).

## 6 Biocompatibility, environmental stability, and toxicological profile of QDs

Despite the excellent optical performance of traditional QDs, their use is limited by heavy-metal toxicity and ion leakage (20). This is where CQDs and GOQDs prove to be a better, biocompatible alternative (23, 39).

### 6.1 Biocompatibility and cellular interactions

The biocompatibility of QDs depends heavily on the surface modification method and the precursor material (6). CDs synthesized from bioactive precursors such as polylysine, melatonin, or metformin have shown minimal cytotoxicity (24). Solubility and stability can be enhanced by surface functionalization, particularly PEGylation (17). Evidence from animal studies also suggests good systemic tolerance, with no notable damage to major organs (24).

### 6.2 Environmental and physicochemical stability

QDs generally exhibit good stability in biological environments, maintaining their optical performance with strong resistance to photobleaching and stable behavior under pH and oxidative changes (4, 20). Protective hybrid platforms like carbon quantum dot-encapsulated metal–organic framework (CQD@MOF) can further improve their stability (18). Degradation of traditional QDs in saliva may expose toxic cores, and their aggregation can reduce the accuracy of their biosensing applications (18, 39).

### 6.3 Toxicological profile and mechanisms of damage

Factors such as particle size, charge, concentration, and the mechanism of uptake determine the toxicity of QDs (20, 39). Toxic effects of conventional cadmium-based quantum dots are primarily linked to cadmium ion release and oxidative stress, which can impair mitochondria, nucleic acids, and other cellular components (39, 40). In contrast, small CDs appear safer, as their clearance is faster through the kidneys, thereby limiting long-term systemic toxicity (14). In an *in-vitro* study, Bismuth Quantum Dot (Bi QD)/ Polydimethylsiloxane (PDMS) coating showed acceptable short-term cytocompatibility, but with a dose-dependent decline in cell viability (41).

The findings of this review position QDs, particularly carbon-based variants, as a multifunctional nanoplatform uniquely suited to the multifactorial nature of periodontitis, capable of integrating diagnostic, therapeutic, regenerative, and implant-related functions within a single material system (2, 8). While QD-based biosensors offer compelling sensitivity for salivary biomarker detection (18) and targeted delivery strategies show site-specific therapeutic promise (19), both await validation under the polymicrobial and biochemically complex conditions of the clinical periodontal environment. Preclinical regenerative data are encouraging, yet remain limited to animal models (2), and the field-wide shift from cadmium-based to carbon-based QDs is a clinical necessity, not merely a refinement (20, 39).

## 7 Regulatory landscape and future scope

There is a major translational barrier when considering the transition of QDs from laboratory synthesis to clinical practice. Lack of harmonized approval pathways, absence of standardized biomarkers, and ethical concerns about privacy, consent, and equitable access limit its translation. The emerging paradigm of personalized nanotheranostics remains in a regulatory grey zone, as it cannot be clearly categorized as either a drug or a device (11). Table 2 delineates the principal challenges to clinical translation, including material cytotoxicity, biocompatibility risk, regulatory barriers, and delivery efficacy limitations, alongside the specific mitigation strategies currently under investigation. Research on QDs is enabling us to move towards more personalized periodontal treatment. Future advances in this field are likely to focus on integrated nanotheranostic platforms that combine high-resolution imaging and targeted, stimuli-responsive drug delivery, leading to more precise diagnosis and on-demand treatment (11, 22). There is growing attention towards greener synthesis strategies that involve natural compounds or pharmacological precursors such as metformin, melatonin, and resveratrol to improve therapeutic relevance (8). The preclinical evaluation method for QDs may be strengthened by including advanced experimental models, such as bone organoids (7). Simultaneous detection of multiple biomarkers may be achieved using multiplex chairside diagnostic platforms to support faster, data-driven clinical decision-making (18).

**Table 2:** Critical challenges and mitigation strategies for clinical translation.

Challenge area	Specific barrier	Underlying mechanism	Mitigation strategy	Impact/clinical necessity
Material Cytotoxicity	Heavy Metal Core Toxicity (CdSe, etc.)	Liberation of free ions due to lattice deterioration, accelerated in the oral environment	Optimized surface coatings (passivation layer); Core engineering	Non-negotiable safety requirement for chronic oral exposure (39, 42)
Biocompatibility Risk	Chronic Local Inflammation/Immune Response	Interaction of surface functional groups or aggregated NPs with host cells and tissues	Transition to inherently biocompatible Carbon Dots (CDs)	CDs receive the highest research attention due to their favorable safety profile (2, 14)
Clinical Feasibility	Regulatory Approval and Adoption	Lack of long-term *in vivo* toxicology and randomized Phase I clinical data	Mandate extended preclinical studies and robust clinical trials	Limited clinical translation remains the major obstacle (3, 4)
Delivery Efficacy	Nanomaterial Aggregation and Stability	Issues related to ionic strength, enzyme activity, and complex crevicular fluid conditions	Functionalization with hydrophilic polymers or peptides for targeting and stability enhancement	Essential for achieving sustained, localized therapeutic windows (20)

## 8 Conclusion

QDs are being recognized as multifunctional nanoplatforms with substantial potential in molecular diagnostics, bioimaging, local drug delivery and periodontal regeneration, which is helping us develop precision based periodontal strategies, but current evidence remains predominantly preclinical; therefore, improvement in physicochemical stability, resolution of toxicity concerns, and standardizing synthesis and characterization will be required to generate long term *in-vivo* and human translational data for its adoption in routine clinical practice.
